# *SZT2* variants associated with partial epilepsy or epileptic encephalopathy and the genotype-phenotype correlation

**DOI:** 10.3389/fnmol.2023.1162408

**Published:** 2023-05-05

**Authors:** Sheng Luo, Xing-Guang Ye, Liang Jin, Huan Li, Yun-Yan He, Bao-Zhu Guan, Liang-Di Gao, Xiao-Yu Liang, Peng-Yu Wang, Xin-Guo Lu, Hong-Jun Yan, Bing-Mei Li, Yong-Jun Chen, Zhi-Gang Liu

**Affiliations:** ^1^Department of Neurology, Institute of Neuroscience, Key Laboratory of Neurogenetics and Channelopathies of Guangdong Province and the Ministry of Education of China, The Second Affiliated Hospital, Guangzhou Medical University, Guangzhou, China; ^2^Department of Pediatrics, Affiliated Foshan Maternity and Child Healthcare Hospital, Southern Medical University, Foshan, China; ^3^Department of Neurology, The Affiliated Nanhua Hospital, Hengyang Medical School, University of South China, Hengyang, China; ^4^Epilepsy Center and Department of Neurology, Shenzhen Children's Hospital, Shenzhen, China; ^5^Epilepsy Center, Guangdong 999 Brain Hospital, Guangzhou, China; ^6^The Second School of Clinical Medicine, Southern Medical University, Guangzhou, China

**Keywords:** *SZT2* gene, partial epilepsy, developmental and epileptic encephalopathy, phenotypic variation, genotype-phenotype correlation

## Abstract

**Background:**

Recessive *SZT2* variants are reported to be associated with developmental and epileptic encephalopathy 18 (DEE-18) and occasionally neurodevelopment abnormalities (NDD) without seizures. This study aims to explore the phenotypic spectrum of *SZT2* and the genotype-phenotype correlation.

**Methods:**

Trios-based whole-exome sequencing was performed in patients with epilepsy. Previously reported *SZT2* mutations were systematically reviewed to analyze the genotype-phenotype correlations.

**Results:**

*SZT2* variants were identified in six unrelated cases with heterogeneous epilepsy, including one *de novo* null variant and five pairs of biallelic variants. These variants had no or low frequencies in controls. All missense variants were predicted to alter the hydrogen bonds with surrounding residues and/or protein stability. The three patients with null variants exhibited DEE. The patients with biallelic null mutations presented severe DEE featured by frequent spasms/tonic seizures and diffuse cortical dysplasia/periventricular nodular heterotopia. The three patients with biallelic missense variants presented mild partial epilepsy with favorable outcomes. Analysis of previously reported cases revealed that patients with biallelic null mutations presented significantly higher frequency of refractory seizures and earlier onset age of seizure than those with biallelic non-null mutations or with biallelic mutations containing one null variant.

**Significance:**

This study suggested that *SZT2* variants were potentially associated with partial epilepsy with favorable outcomes without NDD, expanding the phenotypic spectrum of *SZT2*. The genotype-phenotype correlation helps in understanding the underlying mechanism of phenotypic variation.

## Introduction

The *SZT2* gene (OMIM^*^ 615463) is expressed in the brain, predominantly in the parietal and frontal cortex (Toutzaris et al., 2010). It encodes seizure threshold 2 protein homolog (SZT2), primarily distributing in the lysosome membrane and peroxisome (Peng et al., 2017). In mice, heterozygous knock-out of *Stz2* led to minimal clonic seizures, while the homozygous knock-out causes caused preweaning lethality with incomplete penetrance and maximal tonic hindlimb extension seizures (Frankel et al., 2009). In humans, recessive *SZT2* variants were reported to be associated with developmental and epileptic encephalopathy 18 (DEE-18, OMIM^*^ 615463) and occasionally NDD without seizures (Basel-Vanagaite et al., 2013). The SZT2 protein is the core subunit of the KICSTOR complex, with essential roles in regulating the mechanistic target of rapamycin (mTOR) signal transduction (Wolfson et al., 2017). The mTOR signal pathway plays vital roles in multiple cellular functions, including protein synthesis, cell growth and proliferation, and synaptic plasticity, which will influence neuronal excitability (Meng et al., 2013). Previously, a series of mTOR pathway genes have been identified to be associated with epilepsy and neurodevelopment abnormalities (NDD), including *TSC1, TSC2, PTEN, STRADA, MTOR, AKT3, PIK3CA, RHEB, DEPDC5, NPRL2*, and *NPRL3* (Moloney et al., 2021). The majority of these genes are reported to be associated with a similar phenotypic spectrum, ranging from severe DEE or epilepsy with NDD to mild partial epilepsy (Møller et al., 2016; Liu et al., 2018, 2020; Deng et al., 2019; Jiang et al., 2021). However, it is unknown whether the *SZT2* variants were associated with partial epilepsy, sharing a similar phenotypic spectrum with other mTOR pathway genes.

In this study, trio-based whole-exome sequencing (WES) was performed in patients with epilepsy. A total of 11 *SZT2* variants were identified in six unrelated patients with heterogeneous epilepsy, including DEE in the patients with null variants and partial epilepsy in the patients with compound heterozygous missense variants. To explore the underlying mechanism of phenotypic heterogeneity, we systematically reviewed the previously reported *SZT2* variants and analyzed the correlation between genotype and phenotype.

## Materials and methods

### Patients

The patients were recruited at the Epilepsy Center of the Second Affiliated Hospital of Guangzhou Medical University, Shenzhen Child Hospital, Guangdong 999 Brain Hospital, and Foshan Maternity and Child Healthcare Hospital, through the platform of China Epilepsy Project 1.0. Patients with acquired causes were excluded. Clinical phenotypes of epileptic seizures and epilepsy syndromes were assessed following the criteria of the Commission on Classification and Terminology of the International League Against Epilepsy (1981, 1989, 2001, 2010, 2017). Detailed clinical features were collected, including gender, current age, seizure onset age, seizure type and frequency, outcome, response to antiepileptic drugs, family history, and results from general and neurological examinations. Brain MRI scans were performed to detect brain structure abnormalities. Long-term video electroencephalograms (EEG), including open-close eyes test, hyperventilation, intermittent photic stimulation, and sleep monitoring, were performed. All patients were followed up for at least 1 year.

This study adhered to the principles of the International Committee of Medical Journal Editors concerning patient consent for research or participation and received approval from the ethics committee of the Foshan Maternity and Child Healthcare Hospital (FSFY-MEC-2022-069). Written informed consent was provided by the patient's legal guardians.

### Whole exon sequencing

Blood samples of the probands, their parents, and other available family members were collected. Genomic DNAs were extracted from blood samples using the Qiagen Flexi Gene DNA kit (Qiagen, Hilden, Germany). WES was performed using a NextSeq500 sequencing instrument (Illumina, San Diego, California, USA) following the standard procedures previously described (Wang et al., 2018). The sequencing data were generated by massively parallel sequencing with an average depth of >125x and >98% coverage of the capture region on the chip for obtaining high-quality reads that were mapped to the Genome Reference Consortium Human genome build 37 by Burrows-Wheeler alignment. Variants were called and qualified with the Genome Analysis Toolkit (DePristo et al., 2011). Sanger sequences were used to validate candidate variants.

### Genetic analysis

To derive potentially pathogenic variants, a case-by-case analytical approach was adopted in each case, as described in our previous study (Li et al., 2021; Wang et al., 2021). Initially, the polymorphic variants with a minor allele frequency ≥ 0.005 in the gnomAD database were removed. Potentially disease-causing variants were retained, including frameshift, non-sense, canonical splice site, initiation codon, in-frame, and missense variants. We screened *SZT2* mutations by inheritance origin, including *de novo* mutations, co-segregated mutations, homozygous mutations, and compound heterozygous mutations, which present the genetic difference between the affected child and the parents and thus explain the occurrence of phenotype in a given family (trio). All *SZT2* mutations identified in this study were annotated into the reference transcript NM_015284.

### Analysis of genotype-phenotype correlation

The *SZT2* mutations and related phenotypes were systematically reviewed from the PubMed database (http://www.ncbi.nlm.nih.gov/pubmed/) and the Human Gene Mutation Database (HGMD, http://www.hgmd.cf.ac.uk/ac/index.php) up to November 2022. Null variants were used to define the variant that causes gross malformation of the protein and leads to loss of function (LOF) or haploinsufficiency, such as non-sense, frameshifting, initiation codon, and canonical splice site variants (Richards et al., 2015). The missense and in-frame indel/insert variants were treated as non-null variants.

### Bioinformatic analyses

To evaluate the damaging effect of candidate missense variants, protein modeling was performed by using the Iterative Threading ASSEmbly Refinement software (I-TASSER, https://zhanglab.ccmb.med.umich.edu/I-TASSER/) (Yang and Zhang, 2015). Protein structure changes were visualized and analyzed by PyMOL Molecular Graphics System (Version 2.3.2; Schrödinger, LLC; New York, USA). Protein stability changes of each variant were predicted by the I-Mutant Suite server (gpcr2.biocomp.unibo.it/cgi/predictors/I-Mutant3.0/I-Mutant3.0.cgi) (Capriotti et al., 2005), which was indicated by free energy change (DDG). Negative DDG values indicate abnormally reduced mutant protein stability.

### Statistical analysis

Statistical analyses were performed in R (version 4.0.3). The two-tailed Fisher's exact test was used to compare the difference between groups. *P*-value < 0.05 was considered statistically significant.

## Results

### Identification of *SZT2* variants

**Figure 1:**
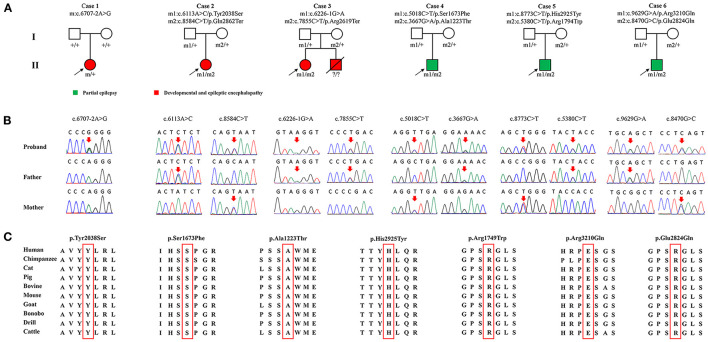
Genetic characterization of the probands. **(A)** Pedigrees of the cases with *SZT2* mutations and their corresponding phenotypes. **(B)** DNA sequence chromatogram of the *SZT2* mutations. Arrows indicated the positions of the mutations. **(C)** The amino acid sequence alignment showed that all seven missense variants were located in residues that are highly conserved across mammals.

A total of 11 *SZT2* variants were identified in six unrelated cases, including one heterozygous mutation (c.6707-2A>G), one pair of biallelic mutations containing one null variant (c.6113A>C/p.Tyr2038Ser & c.8584C>T/p.Gln2862Ter), one pair of biallelic null mutations (c.6226-1G>A & c.7855C>T/p.Arg2619Ter), and three pairs of biallelic missense mutations. (c.5018C>T/p.Ser1673Phe & c.3667G>A/p.Ala1223Thr, c.8773C>T/p.His2925Tyr & c.5380C>T/p.Arg1794Trp, and c.9629G>A/p.Arg3210Gln & c.8470G>C/p.Glu2824Gln) (Figures 1A, B; Table 1). The heterozygous mutation was of *de novo*. The five pairs of compound heterozygous mutations originated from their asymptomatic parents, consistent with Mendelian recessive heredity patterns.

**Table 1:** Clinical characteristics of patients with *SZT2* variants.

**Cases**	**Case 1**	**Case 2**	**Case 3**	**Case 4**	**Case 5**	**Case 6**
Variants (NM_015284)	c.6707-2A>G	p.Tyr2038Ser p.Gln2862Ter	c.6226-1G>A p.Arg2619Ter	p.Ser1673Phe p.Ala1223Thr	p.His2925Tyr p.Arg1794Trp	p.Arg3210Gln p.Glu2824Gln
Sex	F	M	F	M	M	M
Age	4 y	3 yr	2 y	17 y	11 y	7 y
Seizure onset	1 y 11 m	1 y 4 m	1 y	3 y	9 y	4 y
Seizure course	CPS and sGTCS 1–2 times/week; SE twice.	GTCS and sGTCS 1–4 times/day.	CPS, sGTCS, spasms, and tonic seizure 6–10 times/day.	CPS 3–4 times/month.	CPS 3–4 times/month.	sGTCS 2–4 times/year
Prognosis	Refractory	Refractory	Refractory	Seizure free for 6 years	Seizure free for 1 year	Seizure free for 2 years
AEDs	VPA, OXC, TPM, LTG	VPA, OXC, TPM	VPA, TPM, CZP, OXC, LTG, KD	LTG, VPA	LTG, VPA	LTG, VPA
EEG	Background: diffuse slow waves. Interictal: spike and spike-slow mainly in the central-temporal area and occasionally in the frontal and occipital areas.	Background: diffuse slow waves. Interictal: spike and spike-slow in the left temporal region.	Background: diffuse slow waves. Interictal: Spike-slow and poly-spike-slow waves in bilateral frontal and temporal lobes; occasionally generalized poly-spike-slow waves.	Background: normal. Interictal: spike and spike-slow waves in the left temporal lobes at 5-year-old and right frontal and temporal lobes at 15-year-old.	Background: normal. Interictal: spike and spike-slow waves in the bilateral temporal lobe.	Background: normal. Interictal: spike and spike-slow waves in the right frontal and temporal lobes.
Brain MRI	Myelination delay	Myelination delay	Periventricular nodular heterotopia and diffuse cortical dysplasia	Normal	Normal	NA
Development	Severe GDD and ID	Severe GDD and ID	Severe GDD and ID	Normal	Normal	Speech delay
Facial features	High forehead, macrocephaly	High forehead	High forehead, blepharophimosis, and ptosis, thick eyebrows that extend laterally	Normal	Normal	High forehead
Diagnosis	DEE	DEE	DEE	PE	PE	PE

AEDs, anti-epileptic drugs; CPS, complex partial seizures; CZP, clonazepam; DEE, Developmental and epileptic Encephalopathy; EEG, electroencephalogram; F, female; GDD, global development delay; ID, intellectual delay; KD, ketogenic-diet; LEV, levetiracetam; LTG, lamotrigine; m, months; M, male; MRI, magnetic resonance imaging; NA, not available; OXC, oxcarbazepine; PE, partial epilepsy; SE, status epilepticus; sGTCS, secondarily generalized tonic-clonic seizures; TPM, topiramate; VPA, valproic acid; y, years.

The *de novo* heterozygous mutation (c.6707-2A>G) was absent in the gnomAD database. Among variants of biallelic mutations, three null variants and two missense variants (c.6113A>C/p.Tyr2038Ser and c.8470G>C/p.Glu2824Gln) were not presented in controls, while the remaining five missense variants presented low frequencies (MAF < 0.005) in controls of the gnomAD database (Table 2).

**Table 2:** Genetics characteristics of *SZT2* mutations identified in this study.

**Case**	**Nucleotide change**	**Amino acid change**	**Inheritance**	**MAF**	**MAF-EAS**	**SIFT**	**PP2_Var**	**Mutation taster**	**CADD**	**FATHMM_MKL**	**GERP**	**phyloP**	**phastCons**	**SpliceAI**	**ACMG criteria**
Case 1	c.6707-2A>G	-	*De novo*	-	-	-	-	DC (1.000)	D (24.9)	D (0.986)	C (5.99)	C (5.442)	C (1.000)	D (0.99)	P (PVS1+PS2+PM2)
Case 2	c.6113A>C	p.Tyr2038Ser	Paternal	-	-	D (0.000)	PD (0.999)	DC (0.961)	D (28.3)	D (0.988)	C (5.61)	C (9.037)	C (1.000)	-	LP (PM2+PM3+PM5)
	c.8584C>T	p.Gln2862Ter	Maternal	-	-	-	-	DC (1.000)	D (51)	D (0.958)	C (5.36)	C (7.326)	C (1.000)	-	LP (PVS1+PM2)
Case 3	c.6226-1G>A	-	Paternal	-	-	-	-	DC (1.000)	D (25.3)	D (0.934)	C (5.04)	C (7.471)	C (1.000)	D (0.95)	P (PVS1+PM2+PM3)
	c.7855C>T	p.Arg2619Ter	Maternal	-	-	-	-	DC (1.000)	D (43)	D (0.824)	C (4.22)	C (3.307)	C (1.000)	-	P (PVS1+PM2+PM3)
Case 4	c.5018C>T	p.Ser1673Phe	Paternal	3.889 × 10^−5^	5.513 × 10^−4^	D (0.000)	PD (0.999)	DC (0.998)	D (29.5)	D (0.991)	C (5.81)	C (7.307)	C (1.000)	-	US (PM2+PP3)
	c.3667G>A	p.Ala1223Thr	Maternal	2.758 × 10^−4^	3.859 × 10^−3^	T (0.259)	B (0.005)	Po (1.000)	D (23.1)	D (0.915)	C (3.29)	NC (1.414)	C (1.000)	-	US (PM2+PP3)
Case 5	c.8773C>T	p.His2925Tyr	Paternal	3.500 × 10^−4^	4.962 × 10^−3^	T (1.000)	PD (0.997)	DC (0.971)	D (23.3)	D (0.983)	C (5.2)	C (7.458)	C (1.000)	-	US (PM2+PP3)
	c.5380C>T	p.Arg1794Trp	Maternal	1.845 × 10^−4^	1.455 × 10^−3^	T (0.085)	B (0.002)	P (0.996)	D (34)	D (0.961)	C (2.77)	C (2.847)	C (1.000)	-	US (PM2+PP3)
Case 6	c.9629G>A	p.Arg3210Gln	Paternal	5.322 × 10^−6^	7.827 × 10^−5^	T (0.369)	B (0.016)	P (0.981)	D (21.9)	D (0.673)	C (4.64)	C (6.159)	C (1.000)	-	US (PM2+PP3)
	c.8470G>C	p.Glu2824Gln	Maternal	3.982 × 10^−6^	5.438 × 10^−5^	T (0.400)	B (0.227)	DC (0.801)	D (24.2)	D (0.987)	C (5.36)	C (8.536)	C (1.000)	-	US (PM2+PP3)

ACMG, American College of Medical Genetics and Genomics; B, benign; C, conserved; CADD, Combined Annotation Dependent Depletion; D, damaging; DC, disease-causing; Fathmm-MKL, Functional Analysis through Hidden Markov Models–Multiple Kernels Learning; GERP, Genomic Evolutionary Rate Profiling; LP, likely pathogenic; MAF, minor allele frequency of general population from the Genome Aggregation Database; MAF-EAS, minor allele frequency of East Asian population from the Genome Aggregation Database; NC, Non-conserved; P, pathogenic; Po, polymorphism; PD, probably damaging; PM2, absent in population databases; PM5, novel missense change at an amino acid residue where a different pathogenic missense change has been seen before; phastCons, Phylogenetic Analysis with Space/Time models conservation scoring and identification of conserved elements; phyloP, Phylogenetic Analysis with Space/Time models Computation of p-values for conservation or acceleration, either lineage-specific or across all branches; PP2_Var, polyphen2_HVAR; PP3, multiple lines of computational evidence support a deleterious effect on the gene/gene product; PS2, de novo variants (paternity and maternity confirmed); PVS1, predicted null variant in a gene where loss of function (LOF) is a known mechanism of disease; SIFT, Sorting Intolerant From Tolerant; T, tolerable; US, uncertain significance.

According to ACMG guidelines, one missense that constituted biallelic mutation with a null variant and all four null variants were evaluated to be “pathogenic” or “likely pathogenic,” while the other six missense variants that constituted three pairs of compound heterozygous mutations were rated as “uncertain significance.” All missense variants identified in this study were predicted to be “damaging” by at least four *in silico* tools (Table 2). Amino acid sequence alignment indicated that they were located in highly conservated residues across mammals (Figure 1C).

**Figure 2:**
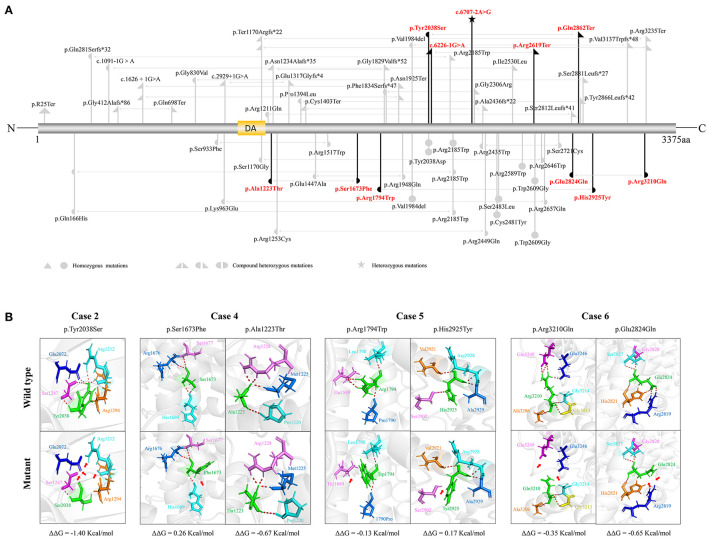
Schematic illustration of variants location, hydrogen bond changes, and protein stability prediction. **(A)** Schematic diagram of SZT2 protein and the localization of the *SZT2* variants identified in this study (red) and reported previously (gray). **(B)** Hydrogen bond changes and DDG values of *SZT2* variants. The red dotted line represented hydrogen bonds. Arrows indicated the positions with hydrogen bond changes. Six of seven missense variants were predicted to alter hydrogen bonds with surrounding residues. The variant p.Ala1223Thr was not predicted to alter hydrogen bonds with surrounding residues but was predicted to decrease the protein stability.

The *SZT2* gene contains 71 exons and encodes a protein that is constituted by a structural domain (1083aa-1189aa), with the function of mediating the interaction with the GATOR1 complex, and other parts with unknown functions. The detailed location of variants in the protein were visualized in Figure 2A.

The molecular effects of the missense variants were analyzed by using the I-TASSER web tool for protein modeling and PyMOL software for visualization. Six of seven missense variants were predicted to alter hydrogen bonds with surrounding residues. The variant p.Ala1223Thr was not predicted to alter hydrogen bonds with surrounding residues but was predicted to decrease the protein stability (Figure 2B).

No pathogenic or likely pathogenic variants in the other epilepsy-related gene were identified in the six patients (Wang et al., 2017).

### Clinical features of the cases with *SZT2* variants

The detailed clinical features of the patients with *SZT2* variants were summarized in Table 1.

**Figure 3:**
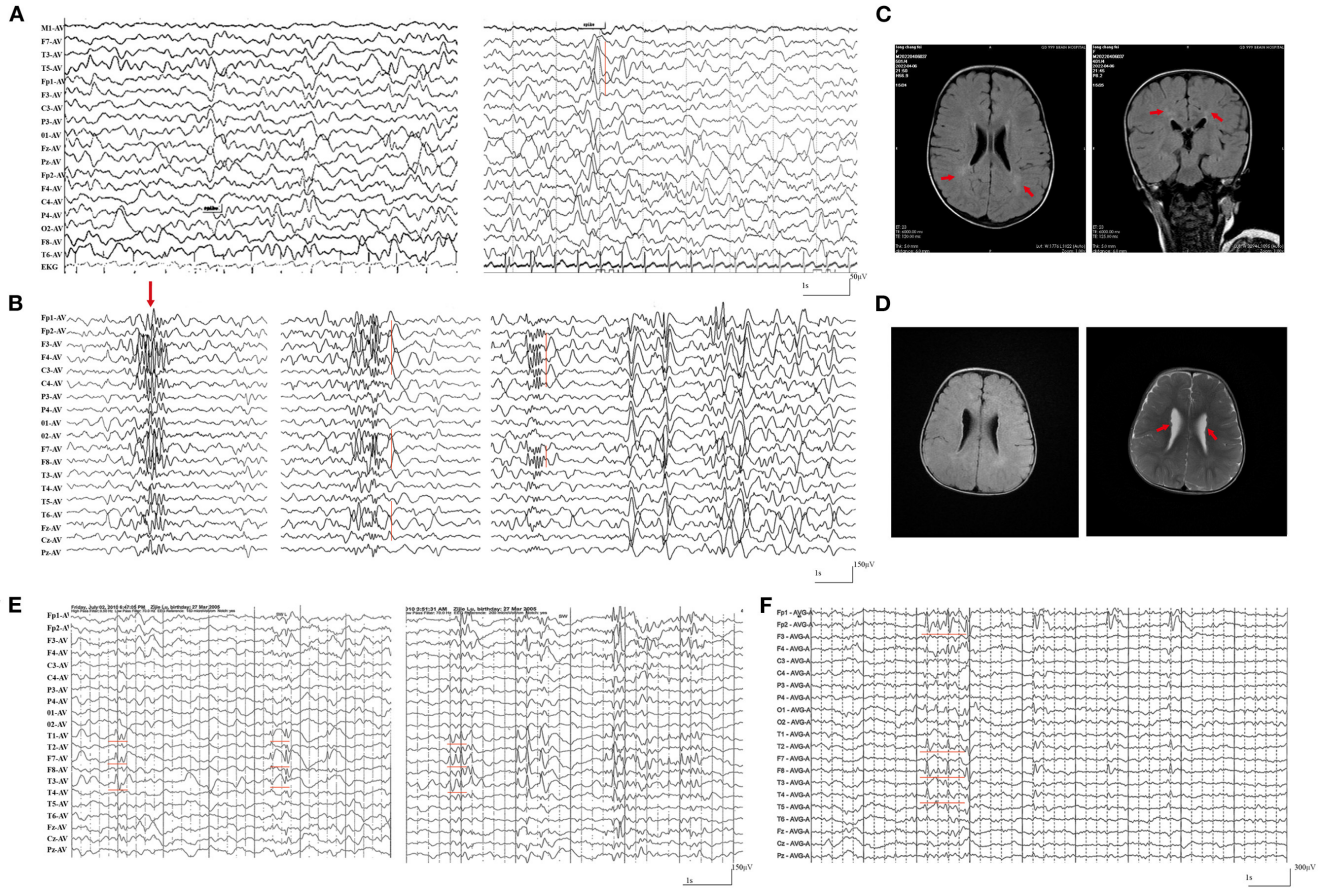
Representative electroencephalography (EEG) and magnetic resonance imaging (MRI) of the cases with *SZT2* mutations. **(A)** Interictal EEG of case 2 at the age of 2-year-old showed general slowing of background (left) and focal sharp waves in the left temporal lobe. **(B)** Interictal EEG of case 3 at the age of 1.5-year-old showed generalized poly-spike waves (left panel) and multifocal poly-spikes/poly-spike-slow waves in the (middle and right panel). **(C)** The MRI of case 2 at the age of 2-year-old showed hyperintensities in the white matter of bilateral periventricular, indicating myelination delay. **(D)** The MRI of case 3 at the age of 1.5-years-old showed diffuse cortical dysplasia and periventricular nodular heterotopia (right panel). **(E)** Interictal EEG of case 4 at 5-year-old showed spike-slow waves in the left temporal lobe. **(F)** Interictal EEG of case 4 at 15-year-old showed spike-slow waves in the right temporal and frontal lobes.

The patients with null *SZT2* variants were all diagnosed with DEE (case 1 case 2, and case 3). They presented daily or weekly seizures with onset ages before 2-year-old. Focal seizures and secondarily generalized tonic-clonic seizures (sGTCS) were the common seizure type. The patients with biallelic null variants (case 3) presented also frequent spasms and tonic seizures. Seizures of the three cases were refractory. The interictal EEG recorded focal or multifocal discharge with backgrounds of diffuse slow waves (Figures 3A, B). The MRI scans detected abnormalities of brain structure, including myelination delay in cases 1 and 2 and diffuse cortical dysplasia/periventricular nodular heterotopia in case 3 (Figures 3C, D). The three patients also exhibited global developmental delays and intellectual disabilities.

The cases with biallelic missense variants were diagnosed with childhood partial epilepsy (cases 4, 5, and 6). They suffered from monthly or yearly focal seizures and/or sGTCS. Seizure-free was achieved with the combined treatment of lamotrigine (LTG) and valproic acid (VPA). The interictal EEG detected focal abnormalities with features of idiopathic epilepsies, including shifting, bilateral, multiple focal discharges with normal backgrounds (Figures 3E, F). The MRI scans detected no brain structural abnormalities. The three patients all exhibited normal neurodevelopment, except case 6 with mild speech delays.

### Genotype-phenotype correlation

**Figure 4:**
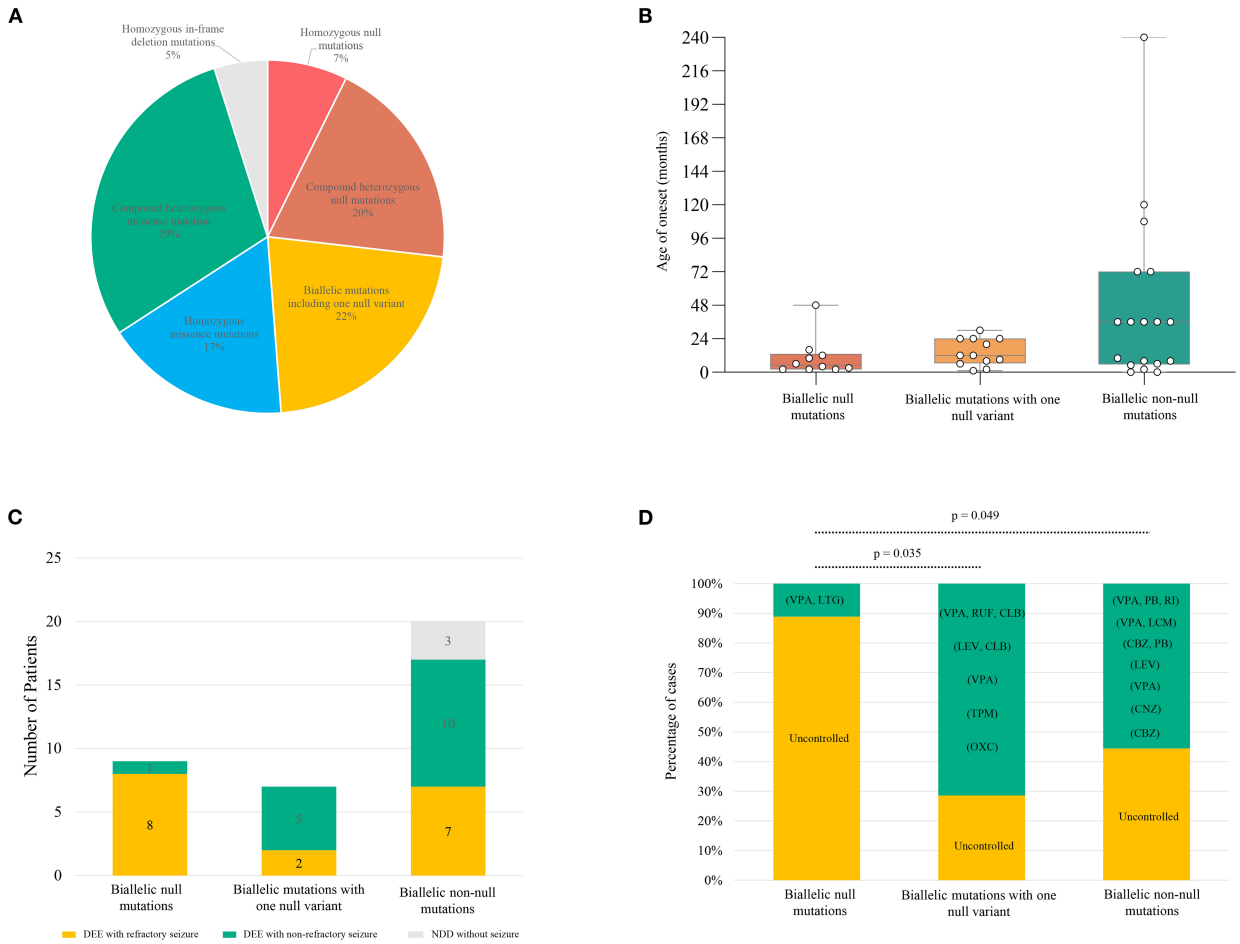
Genotype–phenotype correlation of previously reported *SZT2* mutations. **(A)** The pie chart of the genotype distribution of *SZT2* variants. **(B)** The boxplot of onset ages of the patients with different genotypes. **(C)** The stacked bar chart of phenotypes of the patients with different genotypes. Patients with detailed clinical information were taken into the analysis (*n* = 36). **(D)** The stacked bar chart of drug response of the patients with different genotypes. Patients with biallelic null mutations presented higher percentages of refractory seizures than those with biallelic mutations containing one null variant (8/9 vs. 2/7; *p* = 0.035) and biallelic non-null variants (8/9 vs. 7/17; *p* = 0.049). CBZ, carbamazepine; CLB, clobazam; CNZ, clonazepam; LCM, lacosamide; LEV, levetiracetam; LTG, lamotrigine; OXC, oxcarbazepine; PB, phenobarbital; RUF, rufinamide; TPM, topiramate; VPA, valproic acid.

Previously, a total of 41 pairs of recessive mutations were identified in 50 patients, including 3 homozygous null mutations, 7 pairs of compound heterozygous null mutations, 12 pairs of compound heterozygous mutations containing 1 null variant, 8 homozygous missense mutations, 9 pairs of compound heterozygous missense mutations, 2 homozygous in-frame indel mutations (Figure 4A; Supplementary Table S1). To analyze the relationship between genotype and phenotype, the genotype was classified into: (1) biallelic null mutations, (2) biallelic mutations containing one null variant, (3) and biallelic non-null mutations. The patients with biallelic null mutations had earlier onset ages of seizure with median of 5 months, comparing the patients with biallelic mutations with one null variant (12 months) and biallelic non-null mutations (36 months) (Figure 4B). The patients with biallelic null mutations all presented DEE, with most (8/9) having refractory seizures. Patients with biallelic mutations containing one null variant also exhibited DEE, but with fewer refractory seizures (2/7). Patients with biallelic non-null mutations also showed fewer refractory seizures (7/20), and three patients did not have seizures (3/20) (Figure 4C). Patients with biallelic null mutations presented higher percentages of refractory seizures than those with biallelic mutations containing one null variant (8/9 vs. 2/7; *p* = 0.035) and biallelic non-null variants (8/9 vs. 7/17; *p* = 0.049) (Figure 4D).

## Discussion

In this study, we identified novel *SZT2* variants in six unrelated patients with heterogeneous epilepsy, including one *de novo* null variant and five pairs of biallelic variants. These variants had no or low frequencies in controls. All missense variants were predicted to alter the hydrogen bonding with surrounding residue and/or protein stability. The patients with monoallelic or biallelic null mutations presented severe DEE, while the three patients with biallelic missense mutations exhibited mild partial epilepsy. Analysis of previously reported cases revealed that patients with biallelic null mutations presented significantly higher percentages of refractory seizures and earlier onset ages of seizure than those with other genotypes. This study suggested that *SZT2* variants were potentially associated with partial epilepsy with favorable outcomes without NDD. The genotype-phenotype correlation helps in understanding the underlying mechanism of phenotypic variation.

**Figure 5:**
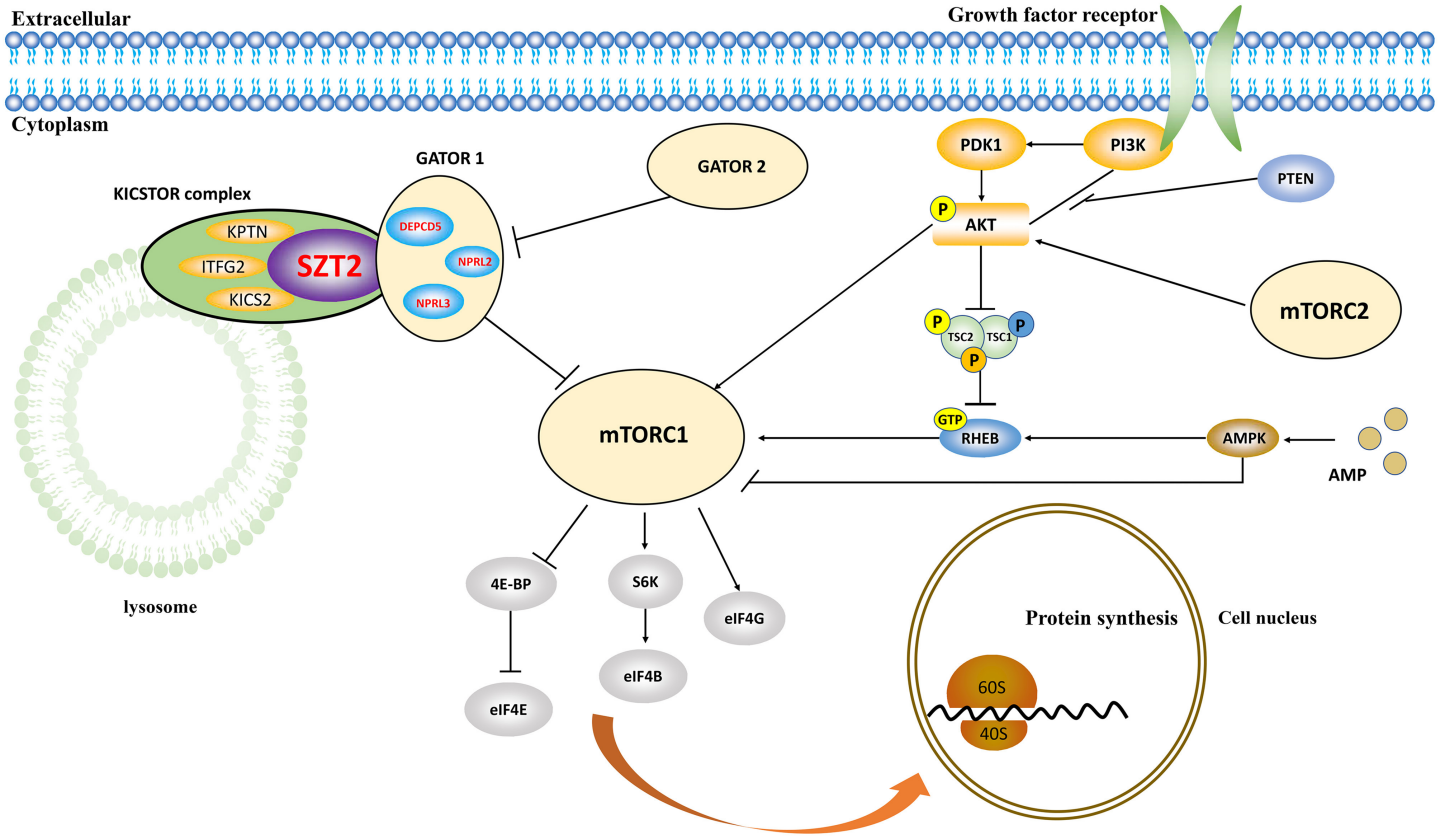
Schematic illustration of the role of *SZT2* in the mTOR pathway. The SZT2 protein is the core subunit of the KICSTOR complex, negatively regulating mTORC1 signaling. It directly interacts with the GATOR1 complex, which was constituted by *DEPDC5, NPRL2*, and *NPRL3*.

The SZT2 protein is the core subunit of the KICSTOR complex (consisting of *KPTN, ITFG2, C12orf66*, and *SZT2*), negatively regulating mTORC1 signaling (Figure 5) (Wolfson et al., 2017). LOF of *SZT2* causes overactivation of mTORC1 signaling, which is one of the hallmarks of epilepsy and brain malformations (Marsan and Baulac, 2018). Experimentally, *Szt2* knockout mice presented spontaneous seizures (Frankel et al., 2009). The variants identified in this study included one heterozygous null variant, one pair of biallelic null mutations, and one pair of biallelic mutations containing one null variant, which were possibly associated with LOF. The six missense variants in the three pairs of biallelic variants were predicted to alter the hydrogen bonding/protein stability and be “damaged” by diverse *in silico* tools, thus being considered to be potentially deleterious. These evidence suggested that LOF may be the pathogenic mechanism for *SZT2*.

The SZT2 protein directly interacts with the GATOR1 complex, which was constituted by *DEPDC5, NPRL2*, and *NPRL3* (Figure 5). These genes were associated with heterogeneous epilepsy (Baldassari et al., 2019), ranging from mild partial epilepsy to severe epileptic encephalopathy (Baldassari et al., 2019; Iffland et al., 2019; Samanta, 2022). It was considered that *SZT2* was associated with a similar phenotypic spectrum, like the three genes. Previously, the *SZT2* variants have been reported to be associated with DEE and occasionally NDD without seizures (Supplementary Table S1). This study identified *SZT2* variants in patients that presented mild partial epilepsy with favorable outcomes without NDD, expanding the phenotypic spectrum of *SZT2*.

In mice, heterozygous knockout of *Szt2* caused minimal clonic seizures, whereas homozygous knockout led to maximal tonic hindlimb extension seizures and preweaning lethality with incomplete penetrance (Frankel et al., 2009). In this study, the patients with null mutations presented DEE; the patient with biallelic null variants presented further severer phenotype featured by frequent spasms/tonic seizures and brain structure abnormalities; patients with biallelic missense variants presented mild partial epilepsy. Similarly, the analysis of previously reported cases showed that patients with biallelic null mutations presented significantly higher percentages of refractory seizures and earlier onset age of seizure than those with other genotypes. These findings suggested a dose-dependent effect of *SZT2*, which is potentially one of the explanations for the phenotypic variation of *SZT2* variants.

To our knowledge, the previously reported *SZT2* mutations were all biallelic variants. This study reported a *de novo* monoallelic variant of *SZT2* (c.6707-2A>G) in a patient with DEE, which was classified as “pathogenic” according to the ACMG criteria. Additionally, the clinical features of the patient coincided highly with DEE-18. This variant was therefore considered to be associated with the phenotype. It is unknown whether other factors, such as dominant negative effects or undetectable intron variants in trans by WES, are involved in the pathogenicity, which warrants further verification.

In the presented study, seizures were refractory in patients with null *SZT2* mutations, whereas seizures-free was achieved in patients with biallelic missense variants by using VPA and LTG. Previously, VPA and LTG were also common effective drugs for patients with *SZT2* mutations, including a seizure-free patient with biallelic null mutations (Figure 4D). It was considered that VPA and LTG may be used as basic therapeutic regimens for patients with *SZT2* mutations, which will be potentially beneficial for patients if an early genetic diagnosis could be made.

This study has several limitations. The pathogenicity of the variants identified in this study was only supported by *in silico* analysis; the detailed functional alteration needs to be experimentally examined. Due to the limitation of WES, potentially disease-causing intron variants or copy number variants may not be detected, especially case 1 with only a heterozygous *SZT2* variant. The genotype-phenotype correlation needs more cases to further elucidate in the future.

In summary, this study suggested that *SZT2* variants were potentially associated with partial epilepsy with favorable outcomes without NDD, expanding the phenotypic spectrum of *SZT2*. The genotype-phenotype correlation and dose-dependent effect help in understanding the underlying mechanism of phenotypic variation.

## Data availability statement

The original contributions presented in the study are included in the article/Supplementary material, further inquiries can be directed to the corresponding authors.

## Ethics statement

The studies involving human participants were reviewed and approved by the Ethics Committee of the Foshan Maternity and Child Healthcare Hospital (FSFY-MEC-2022-069). Written informed consent to participate in this study was provided by the participants' legal guardian/next of kin.

## Author contributions

SL, LJ, X-GY, and Y-JC contributed to the conception of the study, interpretation of clinical data, and drafting of the manuscript. HL, Y-YH, B-ZG, L-DG, X-YL, X-GL, and B-ML examined the patients and participated in the drafting of the manuscript. Y-JC and Z-GL provided a critical review and substantially revised the manuscript. All authors read and approved the manuscript before submitting it to the journal for publication.
