# Supplementary material for: SZT2 variants associated with partial epilepsy or epileptic encephalopathy and the genotype-phenotype correlation
Source: Front Mol Neurosci. 2023 May 5;16:1162408. doi: 10.3389/fnmol.2023.1162408 (PMC10198435; doi:10.3389/fnmol.2023.1162408)
Supplement: Supplementary file 1 [file Table_1.DOCX]

**Table S1.** Characteristics of previously reported cases with *STZ2* mutations.

| **Patient** | **Variants** | **Gender** | **Onset-age** | **Seizure type** | **Therapy** | **Facial and physical features** | **Development** | **EEG** | **MRI** | **Ref** |
| --- | --- | --- | --- | --- | --- | --- | --- | --- | --- | --- |
| 1 | c.73C>T/p.R25* (Hmz) | F | 4y | sGTCS | Refractory | High forehead, down slanting palpebral fissures, ptosis, and arched and laterally extended eyebrows. | Severe DD, No walk, No speech | Isolated spike waves and sharp waves in the right frontopolar and right frontocentral areas; focal epileptic activity with secondary generalization | Thick CC Persistent CSP | [1] |
| 2 | c.498G>T/p.Gln166His  c.6553C>T/p.Arg2185Trp | M | 8m | CPS, sGTCS | Controlled with VPA and LCM | Macrocephaly | Mild ID, DD, ASD, Poor speech | Multifocal small spikes involving mainly frontal and/or occipital regions | Normal | [2] |
| 3 | c.498G>T/p.Gln166His  c.6553C>T/p.Arg2185Trp | M | 4d | CPS, sGTCS, SE | Refractory | Macrocephaly | Severe ID, DD, ASD, No speech, No walk | Multifocal and generalized abnormalities more frontal | Right frontal polymicrogyria | [2] |
| 4 | c.1045delT/p.Ser349Profs*9  c.1891G>A/p.Glu631Lys | M | 6m | GTCS, CPS, ES | NA | NA | Severe ID and DD | NA | Dysmorphic CC, septations in the frontal ventricles, polymicrogyria | [3] |
| 5 | c.2092C>T/p.Gln698*  c.1496G>T/p.Gly412Alafs*86 | M | 2m | Atypical absences, tonic | Refractory | High forehead, down slanting palpebral fissures, ptosis, and arched and laterally extended eyebrows. | Severe DD, No walk, No speech | Highly abnormal | Thick CC Persistent CSP | [1] |
| 6 | c.2732A>G/p.Glu911Gly  c.8640delC/p.Ser2881Hisfs*41 | F | 1m | CPS, spasms | NA | NA | DD | NA | NA | [4] |
| 7 | c.8435delC/p.Ser2812Leufs*41  c.3632G>A/p.Arg1211Gln | F | 2y6m | CPS, tonic | Controlled with VPA, RUF and CLB | Prominent forehead, frontal bossing, down-slanting palpebral fissures, prognathism, normal ear, dental malposition, ligamentous laxity | Severe DD, No speech | Temporal theta waves on the right temporal regions (focal seizures) and bursts of generalized fast poly spikes | Thick CC, right hippocampal atrophy | [5] |
| 8 | c.4209C>A/p.Cys1403*  c.7307_7308insG/p.A2436fs*22 | M | 4m | CPS, tonic, SE | Refractory | High forehead | Severe DD, No speech | Multifocal discharges with partial generalization | Subependymal nodules; shortened CC; widened CSP | [6] |
| 9 | c.5772dupA/p.Asn1925Ter  c.1626 + 1G>A/c.1626 + 1G>A | F | 10m | CPS, clonic, SE | Refractory | High forehead, flattened nasal bridge, hypertelorism | Severe DD and ID | Epileptic discharges originating from L medial temporal area on ictal | Low T1 and high T2 signals in the white matter aged; delayed myelination in the terminal zone | [6] |
| 10 | c.5772dupA/p.Asn1925Ter  c.1626 + 1G>A/c.1626 + 1G>A | M | 1y4m | CPS, tonic, SE | Refractory | High forehead, flattened nasal bridge, hypertelorism | Severe DD, No speech, ID | Epileptic discharges originating from left temporal area on ictal EEG (1 year 6 months) | Subependymal cyst and widened cavum septum pellucidum (14 days after birth); Normal (11 months) | [6] |
| 11 | c.4549C>T/p.Arg1517Trp  c.2798C>T/p.Ser933Phe | F | 4d | CPS, GTCS, tonic, clonic | Controlled with CBZ | High forehead, slightly down-slanting palpebral fissures, and laterally extended eyebrows. | Severe DD, poor speech, no walk | A suppression-burst pattern | Thick CC and persistent CSP | [7] |
| 12 | c.6112T>G/p.Tyr2038Asp(Hmz) | F | NA | Intractable seizures | Refractory | NA | DD, speech delay | NA | NA | [8] |
| 13 | c.6112T>G/p.Tyr2038Asp(Hmz) | F | NA | NA | Refractory | NA | DD, ID | NA | NA | [8] |
| 14 | c.6553C>T/p.Arg2185Trp(Hmz) | M | 10y | CPS | Refractory | High forehead, macrocephaly | DD, speech delay, ASD | Multifocal spikes or spikes and waves predominantly in the frontal lobes | NA | [9] |
| 15 | c.6916G>A/p.Gly2306Arg  c.5499del/p.Phe1834Serfs*47 | F | 1y8m | CPS | Refractory | Macrocephaly with frontal bossing, flattened nasal bridge, hypertelorism | Severe DD, No speech | Characterized by multifocal epileptic form discharges | CC dysgenesis, loss of myelination and progressive atrophy | [10] |
| 16 | c.6958G>C/p.Ala2320Pro  c.8488C>T/p.Arg2830* | NA | NA | NA | NA | NA | NA | NA | NA | [11] |
| 17 | c.7303C>T/p.Arg2435Trp  c.8162C>G/p.Ser2721Cys | F | 8m | Tonic, spasms | Controlled with CNZ | Microcephaly, right Duane anomaly, high-arched palate, and right convergent squint | Bedridden, no speech, severe ID | Multifocal epileptic form discharges | Thick CC, dilated lateral and third ventricles | [12] |
| 18 | c.7303C>T/p.Arg2435Trp(Hmz) | M | 10m | NA | NA | NA | NA | NA | Hyperintensities in bilateral periventricular white matter more in the peri trigonal region. Subcortical white matter hyper | [13] |
| 19 | c.7442G>A/p.Cys2481Tyr(Hmz) | M | 3y | GTCS | Controlled with PB, VPA and risperidone | High forehead | Severe DD, no speech, ASD | Normal | Normal | [14] |
| 20 | c.9368G>A/p.Gly3123Glu(Hmz) | F | 6y | NA | NA | NA | Severe DD, ID, no speech, no walk | Paroxysmal epileptic discharge noted primarily on the right central hemispheres | NA | [15] |
| 21 | c.9703C>T/p.Arg3235*  c.3509_3512del/p.Ter1170Argfs*22 | M | 2m | sGTCS | Controlled with VPA and LTG | Macrocephaly, high forehead, and down-slanted palpebral fissures | Severe DD, no walk, speech delay | Multifocal epileptiform discharges | Thick CC | [16] |
| 22 | c.3497dup/p.Glu1317Glyfs*4  c.2929+1G>A | M | 3m | CPS | Refractory | High forehead | Bedridden, no speech, severe ID | Rhythmic fast waves, low amplitude fast waves, rhythmic slow waves, bursts of spikes and sharp waves | Thick CC, persistent CSP, diffuse brain atrophy | [12] |
| 23 | c.5949_5951delTGT/p.Val1984del  c.4854-2A>G | M | 1y | NA | NA | NA | NA | NA | NA | [17] |
| 24 | c.7341-2A>G(Hmz) | NA | NA | NA | NA | NA | DD | NA | NA | [18] |
| 25 | c.9703C>T/p.Arg3235Ter  c.3509_3512del/p.Thr1170Argfs*22 | NA | NA | NA | NA | NA | NA | NA | NA | [19] |
| 26 | c.3700_3716del17/p.Asn1234Alafs*35  c.5482delC/p.Gly1829Valfs*52 | F | 2m | CPS, sGTCS | Refractory | High forehead, bilateral ptosis, down slanting palpebral fissures, and arched and laterally-extended eyebrows | Bedridden, no speech, severe ID | Isolated spike waves in the left frontocentral areas | Thick CC | [12] |
| 27 | c.4202_4204delTT/p.Phe1401del (Hmz) | M | NA | - | NA | Macrocephaly | Severe DD, speech delay, ID | Normal | Normal | [20] |
| 28 | c.4202_4204delTT/p.Phe1401del (Hmz) | M | NA | - | NA | Macrocephaly | DD, speech delay, moderate ID | Normal | Normal | [20] |
| 29 | c.4202_4204delTT/p.Phe1401del (Hmz) | M | NA | - | NA | Macrocephaly | Speech delay and inattention | NA | Normal | [20] |
| 30 | c.7527delC/p.Ser2510Glnfs*86(Hmz) | NA | NA | NA | NA | NA | NA | NA | NA | [19] |
| 31 | c.8596dupT/p.Tyr2866Leufs*42  c.4181C>T & c.2930-17_2930-3del15insCTCGTG/p.Pro1394Leu & - | F | 2y | sGTCS | Controlled with VPA | High forehead, hypertelorism, macrocephaly | Poor speech, no walk | Focal epileptic discharges background slowing | Thick and short CC | [21] |
| 32 | c.2887A>G/p. Lys963Glu  c.7970G>A/p.Arg2657Gln | M | 8m | CPS | Controlled with VPA | Head circumference of 48 cm (+ 2 standard deviation) | ID, No speech | Slow background rhythm with sharp waves in the right central, parietal, occipital, and temporal areas | A fluid-filled cavity in the midline | [21] |
| 33 | c.3508A>G/p.Ser1170Gly  c.7936C>T/p.Arg2646Trp | F | 2m | Spasms, CPS | Refractory | NA | DD, ID, no speech | Extensive spike and slow-wave complexes or slow-wave complexes with inter-mitten bursts of fast rhythm | A small cyst in the right choroidal fissure | [21] |
| 34 | c.8640_8641insC/p.Ser2881Leufs*27  c.2489G>T/p. Gly830Val | M | 1y | GTCS | Controlled with TPM | NA | DD, ID | Originated from the low-amplitude, fast-wave rhythm that dominated in the left parietal, occipital, and posterior temporal regions | Scaphocephaly and dysplasia of the CC | [21] |
| 35 | c.9368G>A/p.Gly3123Glu(Hmz) | F | 3y | NA | NA | NA | Severe DD and ID, no speech | A background of predominately beta wave rhythm, Paroxysmal epileptic discharge | Prominent extra axial cerebrospinal fluid (CSF) space with wide Sylvian fissure | [15] |
| 36 | c.1496 + 2T > C  c.9055T>C/p.Arg3019Ter | F | 6m | CPS | NA | Macrocephaly, frontal bossing, hypertelorism, microphthalmia, depressed nasal bridge | NA | Developmental epileptic encephalopathy with multifocal epileptiform discharges. | Thick and short CC | [22] |
| 37 | c.7825T>G/p.Trp2609Gly(Hmz) | F | 5m | sGTCS | Controlled with CBZ, PB | Macrocephaly, prominent, forehead, frontal bossing, down slanting palpebral ﬁssures, ptosis | Moderate ID, behavioral disturbances | A long-lasting bilateral seizure with onset over the right central  and parietal regions | Thick CC | [23] |
| 38 | c.7825T>G/p.Trp2609Gly(Hmz) | F | 6m | sGTCS | Refractory | Macrocephaly, prominent forehead, frontal bossing, synophrys of eyebrows, hypertelorism, down slanting palpebral ﬁssures | Moderate ID, behavioral disturbances | A long-lasting bilateral seizure with onset over the right central and parietal regions | CC abnormally shaped | [23] |
| 39 | c.9407_9408dupTG/p.Val3137Trpfs*48  c.5459_5951delTGT/p.Val1984del |  | 2y | sGTCS | Refractory | Macrocephaly | DD, ASD | NA | Non-specific diffusion restriction in the bilateral cerebellar hemispheres | [24] |
| 40 | c.9703CT/p.Arg3235Ter  c.3509_3512delCAGA/p.Thr1170Argfs*22 |  | 2m | sGTCS | Refractory | Macrocephaly | DD, ASD | NA | Right PVNH & abnormal perisylvian gyral configuration; Pineal cyst (13mm); Small pars intermedia cyst | [24] |
| 41 | unknown  c.5459_5951delTGT/p.Val1984del |  | 4y | sGTCS | Controlled with CLB, VPA | Macrocephaly | DD, ASD | NA | None | [24] |
| 42 | c.5459_5951delTGT/p.Val1984del(Hmz) |  | 3y | GTCS | Controlled | Macrocephaly | DD | NA | Ischemic changes (s/p premature delivery and hemorrhage) | [24] |
| 43 | c.2384_5680del/p.His795_His1893del  c.1678G＞T/p.Ala560Ser |  | 2DoL | SGTCS | Refractory | Macrocephaly | DD | NA | Bilateral & multifocal areas of MCD involving both hemispheres. | [24] |
| 44 | c.841delC/p.Gln281Serfs*32  c.6553＞T/p.Arg2185Trp |  | 2y | GTCS | Controlled with OXC | Macrocephaly | DD | NA | Normal | [24] |
| 45 | c.1173_1174del/p.Lys393Glyfs*47  c.4040＞A/p.Arg1347His |  | No Sz | NA | NA | Macrocephaly | DD | NA | Narrowing of the middle third of the Sylvian aqueduct (mild), Myelination delay (mild) | [24] |
| 46 | c.1091-1G＞A  c.7588＞C/p.Ile2530Leu |  | 9m | Absence, GTCS | Controlled with CLB, LEV | Normal | DD | NA | Normal | [24] |
| 47 | c.7346＞A/p.Arg2449Gln  c.3757＞T/p.Arg1253Cys |  | 3y | Absence, GTCS | Controlled with LEV | Frontal bossing, high arched palate | DD | NA | None | [24] |
| 48 | c.7448CT/p.Ser2483Leu(Hmz) |  | 3DoL | GTCS | Controlled | Macrocephaly, thick CC | DD | NA | None | [24] |
| 49 | c.7765C＞T/p.Arg2589Trp  (Hmz) |  | 6y | Myoclonic, absence, GTCS | Refractory | Protruding ears, up slanting palpebral fissures, thick eyebrows, Diastema | Developmental  regression, ID | NA | None | [24] |
| 50 | c.5705T＞C/p.Val1902Ala  c.2887A＞G/p.Lys963Glu | NA | NA | aAS, spasms, FS | NA | NA | GDD, ID | Generalized and multifocal discharges (dominated by the posterior) | White matter dysplasia | [25] |

Abbreviations: aAS, atypical absence seizure; ASD, autistic spectrum disorder; CBZ, carbamazepine; CC, corpus callosum; CPS, complex partial seizures; CLB, clobazam; CNZ, clonazepam; CSP, cavum septum pellucidum; DD, development delay; EEG, electroencephalogram; F, female; FS, febrile seizure; GDD, global developmental delay; GTCS, generalized tonic-clonic Seizure; Hmz, homozygous; ID, intellectual delay; KD, ketogenic-diet; LCM, lacosamide; LEV, levetiracetam; LTG, lamotrigine; m, months; M, male; MRI, magnetic resonance imaging; NA, not available; OXC, oxcarbazepine; PB, phenobarbital; PVNH, periventricular nodular heterotopia; RUF, rufinamide; SE, status epilepticus; Sz, seizure; sGTCS, secondarily generalized tonic-clonic seizures; TPM, topiramate; VPA, valproic acid; y, years.

1. Basel-Vanagaite L, Hershkovitz T, Heyman E, Raspall-Chaure M, Kakar N, Smirin-Yosef P, Vila-Pueyo M, Kornreich L, Thiele H, Bode H *et al*: **Biallelic SZT2 mutations cause infantile encephalopathy with epilepsy and dysmorphic corpus callosum**. *Am J Hum Genet* 2013, **93**(3):524-529.

2. Domingues FS, König E, Schwienbacher C, Volpato CB, Picard A, Cantaloni C, Mascalzoni D, Lackner P, Heimbach A, Hoffmann P *et al*: **Compound heterozygous SZT2 mutations in two siblings with early-onset epilepsy, intellectual disability and macrocephaly**. *Seizure* 2019, **66**:81-85.

3. Papuc SM, Abela L, Steindl K, Begemann A, Simmons TL, Schmitt B, Zweier M, Oneda B, Socher E, Crowther LM *et al*: **The role of recessive inheritance in early-onset epileptic encephalopathies: a combined whole-exome sequencing and copy number study**. *European journal of human genetics : EJHG* 2019, **27**(3):408-421.

4. Rochtus A, Olson HE, Smith L, Keith LG, El Achkar C, Taylor A, Mahida S, Park M, Kelly M, Shain C *et al*: **Genetic diagnoses in epilepsy: The impact of dynamic exome analysis in a pediatric cohort**. *Epilepsia* 2020, **61**(2):249-258.

5. Iodice A, Spagnoli C, Frattini D, Salerno GG, Rizzi S, Fusco C: **Biallelic SZT2 mutation with early onset of focal status epilepticus: Useful diagnostic clues other than epilepsy, intellectual disability and macrocephaly**. *Seizure* 2019, **69**:296-297.

6. Sun X, Zhong X, Li T: **Novel SZT2 mutations in three patients with developmental and epileptic encephalopathies**. *Mol Genet Genomic Med* 2019, **7**(9):e926.

7. Tanaka R, Takahashi S, Kuroda M, Takeguchi R, Suzuki N, Makita Y, Narumi-Kishimoto Y, Kaname T: **Biallelic SZT2 variants in a child with developmental and epileptic encephalopathy**. *Epileptic Disord* 2020, **22**(4):501-505.

8. Monies D, Abouelhoda M, Assoum M, Moghrabi N, Rafiullah R, Almontashiri N, Alowain M, Alzaidan H, Alsayed M, Subhani S *et al*: **Lessons Learned from Large-Scale, First-Tier Clinical Exome Sequencing in a Highly Consanguineous Population**. *Am J Hum Genet* 2019, **105**(4):879.

9. Imaizumi T, Kumakura A, Yamamoto-Shimojima K, Ondo Y, Yamamoto T: **Identification of a rare homozygous SZT2 variant due to uniparental disomy in a patient with a neurodevelopmental disorder**. *Intractable & rare diseases research* 2018, **7**(4):245-250.

10. Pizzino A, Whitehead M, Sabet Rasekh P, Murphy J, Helman G, Bloom M, Evans SH, Murnick JG, Conry J, Taft RJ *et al*: **Mutations in SZT2 result in early-onset epileptic encephalopathy and leukoencephalopathy**. *Am J Med Genet A* 2018, **176**(6):1443-1448.

11. Hamdan FF, Myers CT, Cossette P, Lemay P, Spiegelman D, Laporte AD, Nassif C, Diallo O, Monlong J, Cadieux-Dion M *et al*: **High Rate of Recurrent De Novo Mutations in Developmental and Epileptic Encephalopathies**. *Am J Hum Genet* 2017, **101**(5):664-685.

12. Tsuchida N, Nakashima M, Miyauchi A, Yoshitomi S, Kimizu T, Ganesan V, Teik KW, Ch'ng GS, Kato M, Mizuguchi T *et al*: **Novel biallelic SZT2 mutations in 3 cases of early-onset epileptic encephalopathy**. *Clin Genet* 2018, **93**(2):266-274.

13. Parayil Sankaran B, Nagappa M, Chiplunkar S, Kothari S, Govindaraj P, Sinha S, Taly AB: **Leukodystrophies and Genetic Leukoencephalopathies in Children Specified by Exome Sequencing in an Expanded Gene Panel**. *Journal of child neurology* 2020, **35**(7):433-441.

14. Kariminejad A, Yazdan H, Rahimian E, Kalhor Z, Fattahi Z, Zonooz MF, Najmabadi H, Ashrafi M: **SZT2 mutation in a boy with intellectual disability, seizures and autistic features**. *Eur J Med Genet* 2019, **62**(9):103556.

15. Naseer MI, Alwasiyah MK, Abdulkareem AA, Bajammal RA, Trujillo C, Abu-Elmagd M, Jafri MA, Chaudhary AG, Al-Qahtani MH: **A novel homozygous mutation in SZT2 gene in Saudi family with developmental delay, macrocephaly and epilepsy**. *Genes Genomics* 2018, **40**(11):1149-1155.

16. Venkatesan C, Angle B, Millichap JJ: **Early-life epileptic encephalopathy secondary to SZT2 pathogenic recessive variants**. *Epileptic Disord* 2016, **18**(2):195-200.

17. Ji J, Shen L, Bootwalla M, Quindipan C, Tatarinova T, Maglinte DT, Buckley J, Raca G, Saitta SC, Biegel JA *et al*: **A semiautomated whole-exome sequencing workflow leads to increased diagnostic yield and identification of novel candidate variants**. *Cold Spring Harbor molecular case studies* 2019, **5**(2).

18. Nair P, Sabbagh S, Mansour H, Fawaz A, Hmaimess G, Noun P, Dagher R, Megarbane H, Hana S, Alame S *et al*: **Contribution of next generation sequencing in pediatric practice in Lebanon. A Study on 213 cases**. *Mol Genet Genomic Med* 2018, **6**(6):1041-1052.

19. Retterer K, Juusola J, Cho MT, Vitazka P, Millan F, Gibellini F, Vertino-Bell A, Smaoui N, Neidich J, Monaghan KG *et al*: **Clinical application of whole-exome sequencing across clinical indications**. *Genet Med* 2016, **18**(7):696-704.

20. Falcone M, Yariz KO, Ross DB, Foster J, 2nd, Menendez I, Tekin M: **An amino acid deletion inSZT2 in a family with non-syndromic intellectual disability**. *PLoS One* 2013, **8**(12):e82810.

21. Nakamura Y, Togawa Y, Okuno Y, Muramatsu H, Nakabayashi K, Kuroki Y, Ieda D, Hori I, Negishi Y, Togawa T *et al*: **Biallelic mutations in SZT2 cause a discernible clinical entity with epilepsy, developmental delay, macrocephaly and a dysmorphic corpus callosum**. *Brain & development* 2018, **40**(2):134-139.

22. Hong SY, Yang JJ, Li SY, Lee IC: **A Wide Spectrum of Genetic Disorders Causing Severe Childhood Epilepsy in Taiwan: A Case Series of Ultrarare Genetic Cause and Novel Mutation Analysis in a Pilot Study**. *J Pers Med* 2020, **10**(4).

23. Trivisano M, Rivera M, Terracciano A, Ciolfi A, Napolitano A, Pepi C, Calabrese C, Digilio MC, Tartaglia M, Curatolo P *et al*: **Developmental and epileptic encephalopathy due to SZT2 genomic variants: Emerging features of a syndromic condition**. *Epilepsy Behav* 2020, **108**:107097.

24. Calhoun JD, Aziz MC, Happ HC, Gunti J, Gleason C, Mohamed N, Zeng K, Hiller M, Bryant E, Mithal DS *et al*: **mTORC1 functional assay reveals SZT2 loss-of-function variants and a founder in-frame deletion**. *Brain* 2022, **145**(6):1939-1948.

25. Niu Y, Gong P, Jiao X, Xu Z, Zhang Y, Yang Z: **Genetic and phenotypic spectrum of Chinese patients with epilepsy and photosensitivity**. *Front Neurol* 2022, **13**:907228.
